# The temporal relation between pain and fatigue in individuals receiving treatment for chronic musculoskeletal pain

**DOI:** 10.1186/s12891-022-05162-7

**Published:** 2022-03-08

**Authors:** Keiko Yamada, Heather Adams, Tamra Ellis, Robyn Clark, Craig Sully, Christian Lariviere, Michael JL Sullivan

**Affiliations:** 1 Department of Psychology, McGill University, 2001 McGill College, QC H3A 1G1 Montreal, Canada; 2 Department of Anesthesiology and Pain Medicine, Juntendo University Faculty of Medicine, Tokyo, Japan; 3 University Centre for Research and Disability, Halifax, Nova Scotia Canada; 4 Centre for Rehabilitation and Health, Toronto, ON Canada; 5 Kootenay Health Services, Nelson, BC Canada; 6 l’Institut de recherche Robert-Sauvé en santé et en sécurité du travail (IRSST), Montreal, QC Canada

**Keywords:** Pain, Fatigue, Musculoskeletal, Rehabilitation

## Abstract

**Background:**

Numerous investigations have revealed significant relations between pain and fatigue in individuals with persistent pain conditions. However, the direction of influence between pain and fatigue remains unclear. Shortcomings of design and analytic approaches used in previous research limit the nature of conclusions that can be drawn about possible causal or directional relations between pain and fatigue. The present study investigated the temporal relation between changes in pain and changes in fatigue in individuals with musculoskeletal pain enrolled in a 10-week behavioral activation intervention. On the basis of previous findings, it was hypothesized that analyses would support a bi-directional relation between pain and fatigue.

**Methods:**

The study sample consisted of 104 individuals with chronic musculoskeletal pain participating in a 10-week standardized rehabilitation intervention. Measures of pain intensity and fatigue were completed pre-, mid-, and post-treatment. The three-wave data panel permitted examination of the direction of influence between pain and fatigue through the course of the intervention. A random-intercept cross-lagged panel model (RI-CLPM) was used to examine the temporal relation between pain and fatigue.

**Results:**

Consistent with previous research, cross-sectional analyses of pre-treatment data revealed significant correlations between measures of pain and fatigue. Significant reductions in pain and fatigue were observed through the course of treatment (*d* = 0.33 and *d* = 0.66, *p* < .001, respectively). RI-CLPM revealed that pain severity predicted later fatigue (pre to mid-treatment standardized path coefficient (β) = 0.55, *p* = 0.02; mid to post-treatment β = 0.36, *p* = 0.001); however, fatigue did not predict later pain severity.

**Conclusions:**

Discussion addresses the processes that might underlie the temporal relation between pain and fatigue. Clinical implications of the findings are also discussed.

## Background

Persistent fatigue is a frequent complaint of individuals with chronic pain [1]. Persistent fatigue has been broadly defined as overwhelming sense of tiredness, lack of energy and a feeling of exhaustion that is unrelated to the recent activity [2]. A relation between pain intensity and persistent fatigue has been reported in several populations where pain is a significant symptom including osteoarthritis [3], rheumatoid arthritis [4], fibromyalgia [5], cancer [6], headaches [7], and low back pain [8]. Approximately half of individuals with chronic pain report fatigue as their most debilitating symptom [2, 9].

Research conducted to date suggests that there might be a temporal relation between pain and fatigue [10]. Fishbain et al. [1] reported that in 5 of the 6 prospective studies they reviewed, the development of fatigue occurred after pain onset, suggesting that pain might be causally related to fatigue. There are also indications that symptoms of fatigue might precede the onset of pain. Siivola et al. [11] reported that symptoms of fatigue were prospectively associated with the onset of musculoskeletal pain in a sample of healthy young adults. Halder et al. [12] reported that symptoms of fatigue were prospectively associated with the onset of abdominal pain in a sample of patients drawn from a primary care registry.

The pattern of findings that has emerged from previous research supports a relation between pain and fatigue but is limited in its implications for possible causal relations between pain and fatigue. Demonstrating a prospective relation between pain and fatigue does not necessarily point to causal mechanisms. Prospective correlations can be influenced by extraneous sources of variance from auto-correlations (i.e., associations between the same variable measured at different times) and synchronous correlations (i.e., associations between different variables measured at the same time).

Analytic approaches that have been used in previous research, such as regression analyses, cross-lagged panel or path analytic procedures, account only for group-level changes between variables. Research suggests that failing to account for ‘individual-level’ changes in temporally-related variables increases the probability of Type I error, and consequently, can lead to erroneous conclusions [13]. It has been suggested that causal relations between temporally-related variables can best be addressed by analytic procedures that account for individual-level changes in variables of interest in addition to group-level changes [14].

The purpose of the present study was to examine the temporal relation between pain and fatigue in individuals with musculoskeletal conditions who were enrolled in a standardized rehabilitation intervention [14]. Examination of the direction of influence between changes in pain and changes in fatigue through the course of a treatment program could have important clinical implications. Findings might point to potential causal or functional associations between pain and fatigue and in turn, play a role in decisions relevant to the choice of key targets of intervention. Treatments specifically designed to target causal or antecedent variables associated with clinical outcomes could lead to the development of intervention approaches that might contribute to more positive recovery outcomes for individuals with persistent pain conditions.

A random-intercept cross-lagged panel model (RI-CLPM) was used to clarify the direction of influence between changes in pain and changes in fatigue. RI-CLPM is an extension of traditional CLPM permitting isolation of variance attributable to individual-level change [14]. Like traditional CLPM, RI-CLPM also controls for the potential inflation effects of auto- and synchronous relations among variables. RI-CLPM is considered a preferred analysis particularly in cases where variables might be expected to show trait-like stability, such as would be the case with chronic pain and associated fatigue [14]. Based on previous research, it was hypothesized that the results of analyses would support a bi-directional relation between pain and fatigue.

## Methods

### Participants

The study sample consisted of 104 consecutive referrals to an occupational rehabilitation service in Ontario, Canada. All participants were on sick leave and were receiving temporary salary indemnity from an injury/disability insurer at the time of referral. All participants had been employed full time prior to the current period of absence from work. For all participants, musculoskeletal injury (i.e., strain/sprain) to the back or neck was the primary basis for work absence and salary indemnity. On the basis of the nature of participants’ work injury and the time elapsed since injury (> 3 months), participants’ clinical presentation would be consistent with an ICD-11 diagnosis of *chronic primary musculoskeletal pain* [15].

## Measures

### Pain severity

The McGill Pain Questionnaire Short-Form (MPQ-SF) was used to measure pain severity (Melzack, 1987). Participants rated their current pain experience according to 11 sensory and 4 affective pain descriptors on an intensity scale with the anchors (0) *none*, (1) *mild*, (2) *moderate*, or (3) *severe*. Scores can range from 0 to 45 where higher scores represent more intense pain. The measure has been shown to be reliable and valid in various clinical populations [16, 17].

### Fatigue

The Fatigue Measure (FM) was used to assess fatigue [18]. The FM consists of 5 items assessing different symptoms of persistent fatigue. Ratings are made on a 3-point frequency scale with the anchors (0) *never*, (1) *sometimes* and (2) *often*. Scores on the measure of fatigue can range from 0 and 10. Research has supported the reliability and validity of the FM in individuals with musculoskeletal pain [18]. In the present sample, Cronbach’s alpha for the fatigue measure was 0.78.

## Procedure

This study was approved by Institutional Review Board of McGill University in accordance with the Declaration of Helsinki. The study sample was drawn from the de-identified clinical files of individuals who were enrolled in a 10-week standardized risk-targeted behavioural activation intervention [19]. Measures of pain severity and fatigue were completed pre-treatment (T0), mid-treatment (T1) and treatment termination (T2).

Participation in risk-targeted behavioral activation has been associated with clinically significant reductions in pain and fatigue [18]. The intervention was offered as a complement to any other pharmacological or physical interventions that might have been prescribed for symptom management. All participants received treatment between January and December, 2017. Data were extracted in May of 2018.

The sample comprised a subset (104/117) of participants described in a previous publication addressing the role of fatigue as a determinant of occupational disability [18]. The subset consisted of participants for whom complete data on pain and fatigue were available at three time points to permit cross-lagged analyses.

### Intervention: risk-targeted behavioral activation

Clinicians met with participants once per week for a total of 10 weeks. Participants first viewed an introductory video that provided them with information about the disabling effects of persistent pain and oriented them to the procedures and objectives of treatment. Guided disclosure and validation techniques were used to foster the development of the working alliance. Behavioral activation focused on goal setting, maximizing success and achievement experiences, and resumption of life role activities [20]. Activity planning took the form of graduated re-engagement in activities that were discontinued following work injury. In addition to techniques designed to increase participants’ involvement in purposeful activities of daily living, behavioural activation was supplemented by risk-targeted techniques designed to yield reductions in pain-related psychosocial risk factors including catastrophic thinking, perceived injustice and disability beliefs [19]. Education, thought monitoring, re-appraisal, problem-solving and progress reinforcement were used to target catastrophic thinking, perceived injustice and disability beliefs.

Clinicians were occupational therapists who had been trained to competency in the risk-targeted behavioral activation intervention. All clinicians were supervised by a senior clinician to ensure fidelity to the standardized treatment protocol. The risk-targeted intervention used in the present study is described in more detail elsewhere [19].

## Data analytic approach

An a priori sample size calculator for structural equation modeling was used to estimate sample size [21]. With 6 observed variables and 7 latent variables, an anticipated large effect size, and power set at 0.80, a sample size of 104 meets criterion for sampling adequacy for detecting a significant effect (*p* < 0.05). The assumption of large effect size is supported by previous research on the relation between pain and fatigue [18].

T-tests for independent samples were used to compare women and men on continuous variables and chi square analyses were used to compare groups on categorical variables. Pearson correlations were used to examine relations among variables at time of admission, mid- and post-treatment. T-tests for paired variables were used to examine treatment-related changes in fatigue, pain, and depressive symptoms. Intra-class correlation (ICC) using a two-way random effects model was calculated for variables. ICC can be defined as the proportion of the variance explained by differences between participants. RI-CLPM was used to examine the temporal associations between treatment-related changes in symptoms of pain and fatigue following the methods introduced by Hamaker et al. [14]. One of the advantages of RI-CLPM over other approaches to time-series or path analyses is that RI-CLPM isolates within-person associations between two (or more) variables from between-person associations attributable to trait-like characteristics [22]. RI-CLPM allows parsing the part of the variability of a measure that is invariant, trait-like, from genuine autoregressive stability, which results in a more precise estimation of the cross-lagged relationships. Model fitting indices included the comparative fit index (CFI), the Tucker–Lewis index (TLI), the root mean square error of approximation (RMSEA) with 90% confidence interval (CI), and the standardized root-mean-square residual (SRMR) [23].

T-tests for paired variables and Pearson’s correlations were conducted with SAS version 9.4 (SAS Institute Inc., Cary, NC, USA), ICC was calculated with IBM SPSS statistics version 24, and the RI-CLPM was conducted with M-Plus version 8.4.

## Results

### Sample characteristics

Means and standard deviations on all study variables are presented stratified by sex in Table 1. The majority of the participants (62.5%) were married or co-habiting and had completed at least 12 years of schooling (98.1%). Median pain duration for the study sample was approximately 1 year. The majority of participants were using some form of analgesic for pain control. Approximately 10% of the sample was concurrently receiving another form of rehabilitation treatment (e.g., physiotherapy, occupational therapy, psychotherapy). Based on test scores at the time of admission, the study sample would be characterized as experiencing moderate to severe symptoms of pain and fatigue [24].

**Table 1:** Sample characteristics at the time of admission

	Men		Women			
	*N* = 41		*N* = 63			
	Mean	SD	Mean	SD		*P*-value
Age, years	44.7	8.0	45.1	8.0		0.83
	n	%	n	%		
Marital Status						
Single	10	24.4%	13	20.6%		0.61
Married or co-habiting	26	63.4%	39	61.9%		0.46
Separated or divorced	4	9.8%	10	15.9%		
Widowed	1	2.3%	1	1.6%		
Education						
Less than high school	2	4.9%	0	0.0%		
High school/Trade school	13	31.7%	16	25.4%		0.48
College	13	31.7%	37	58.7%		0.007
University	12	29.3%	10	15.9%		0.10
Missing	1	2.4%	0	0.0%		
Pain Duration						0.88
3 – 6 months	4	9.8%	8	12.7%		
7 – 12 months	15	36.6%	21	33.3%		
12 – 24 months	22	53.7%	34	54.0%		
Medication						
NSAIDs	26	63.4%	32	50.8%		0.21
Opioid	6	14.6%	9	14.3%		0.96
Antidepressant	8	19.5%	7	11.1%		0.14
Secondary treatment						
Physiotherapy	3	7.3%	1	1.6%		
Occupational therapy	3	7.3%	5	7.9%		
Psychotherapy	0	0.0%	1	1.6%		
	Mean	SD	Range Mean	SD Range		
MPQ-SF (0 – 45)	17.6	8.7	(6 – 44) 19.0		9.2 (7 – 42)	0.40
FM (0 – 10)	7.4	2.3	(0—10) 7.7		2.0 (0 – 10)	0.47

*N* = 104. *FM* Fatigue Measure, *MPQ* McGill Pain Questionnaire – Short Form, *NSAIDS* Nonsteroidal anti-inflammatory drug, *SD* Standard Deviation

## Pain severity, fatigue: cross-sectional relations

Consistent with previous research [25], pain severity was significantly correlated with fatigue at admission (Table 2). Pain severity and fatigue were also significantly correlated at mid- and post-treatment, *r* = 0.34, *p* < 0.001, *r* = 0.57, *p* < 0.001, respectively.

**Table 2:** Zero-order correlations between pain and fatigue at pre-, mid-, and post-treatment

	1	2	3	4	5
1 MPQ-PRI0	-	-	-	-	-
2 MPQ-PRI1	0.67***	-	-	-	-
3 MPQ-PRI2	0.47***	0.70***	-	-	-
4 FM0	0.39***	0.34***	0.27**	-	-
5 FM1	0.34***	0.34***	0.33***	0.54***	-
6 FM2	0.21*	0.38***	0.57***	0.50***	0.59***

*N* = 104

*FM* Fatigue Measure, *MPQ-PRI* McGill Pain Questionnaire-Pain Rating Index

^*^ *p* < 0.05, ** *p* < 0.01, *** *p* < 0.001. Subscripts: 0 = pre-treatment, 1 = mid-treatment, 2 = post-treatment

### Treatment-related changes in dependent variables

As shown on Table 3, t-tests for paired samples (pre- to mid-, mid- to post-, and pre- to post-treatment) were conducted to examine treatment-related changes in scores on measures of pain and fatigue symptom severity. Participation in the treatment program (pre- to post-treatment) was associated with significant reductions in pain severity, t (102) = 3.3, *p* < 0.001. *d* = 0.33 (95% CI, 0.13–0.52) and fatigue, t (102) = 6.7, p < 0.001, *d* = 0.66 (95% CI. 0.45–0.87). From pre- to mid treatment, 50% of participants showed a change in their fatigue score of 2 points or greater, and 55% of participants showed a change in their MPQ = PRI score of 5 points or greater. From mid- to post-treatment, 40% of participants showed a change in their fatigue score of 2 points or greater, and 52% of participants showed a change in their MPQ = PRI score of 5 points or greater.

**Table 3:** Means Scores for Pain and Fatigue at Pre-, Mid-, and Post-Treatment

**MPQ-PRI (0 – 45)**	**Pre-treatment (T0)**	**Mid-treatment** **(T1)**	**Post-treatment** **(T2)**	
**Mean (SD)**	18.5 (9.0)	17.1 (10.0)	15.1 (10.8)	
** pre- to mid-**				
*p* value		0.07		
Cohen's d (95% CI)		0.18 (-0.01–0.38)		
** mid- to post-**				
*p* value			0.02	
Cohen's d (95% CI)			0.24 (0.05–0.44)	
** pre- to post-**				
*p* value				< 0.001
Cohen's d (95% CI)				0.33 (0.13–0.52)
**FM (0 – 10)**	**Pre-treatment** **(T0)**	**Mid-treatment** **(T1)**	**Post-treatment** **(T2)**	
**Mean (SD)**	7.6 (2.1)	6.6 (2.4)	5.9 (2.9)	
** pre- to mid-**				
*p* value		< 0.001		
Cohen's d (95% CI)		0.49 (0.28–0.69)		
** mid- to post-**				
*p* value			0.01	
Cohen's d (95% CI)			0.26 (0.06–0.45)	
** pre- to post-**				
*p* value				< 0.001
Cohen's d (95% CI)				0.66 (0.45–0.87)

*N* = 104

*CI* Confidence Interval, *FM* Fatigue Measure, *MPQ-PRI* McGill Pain Questionnaire-Pain Rating Index, *SD* standard deviation

### Random Intercept Cross-Lagged Panel Model (RI-CLPM) between pain and fatigue

**Fig. 1:**
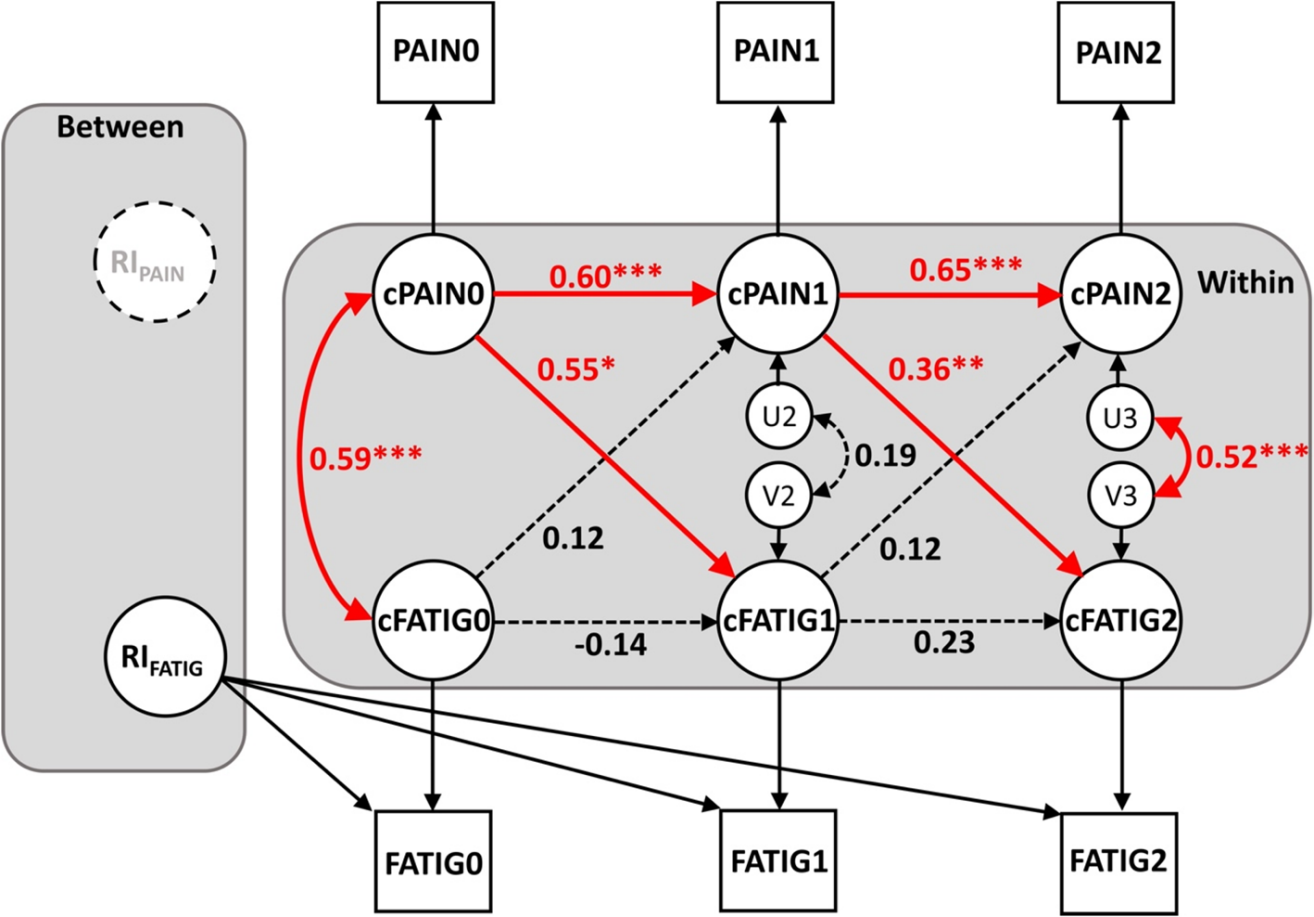
Random intercepts cross-lagged panel model linking pain and fatigue. Note: N=104. Model fit indices: comparative fit index (CFI) = 1.00, Tucker-Levis index (TLI) = 1.00, root mean square error of approximation (RMSEA) < 0.001 (90% confidence interval: 0.00-0.15), standardized root-mean-square residual (SRMR) = 0.03

The results of the RI-CLPM for the temporal association between pain and fatigue, for within- and between-person effects are presented in Fig. 1; standardized estimates of all paths in RI-CLPM are shown in Table 4. Variance and covariance of random-intercept MPQ-PRI were set to zero because the random-intercept for the MPQ-PRI was not significant. For fatigue, the intra-class correlation (ICC) was 0.53 (95% CI, 0.42–0.64); differences in fatigue between participants (i.e., between-person variance) explained 53% of the variance in fatigue across the three measurement points, and within-person variance explained the remaining 47% of the variance across measurement points.

**Table 4:** Random intercepts cross-lagged panel path coefficients

Parameter	β	SE	*P*-value
**Within-person (state-like)**			
** Autoregressive paths**			
MPQ-PRI_0_ → MPQ-PRI_1_	0.60	0.1	< 0.001
MPQ-PRI_1_ → MPQ-PRI_2_	0.65	0.1	< 0.001
FM_0_ → FM_1_	-0.14	0.3	0.63
FM_1_ → FM_2_	0.23	0.1	0.08
** Correlations**			
MPQ-PRI_0_ – FM_0_	0.59	0.1	< 0.001
MPQ-PRI_1_ – FM_1_	0.19	0.1	0.18
MPQ-PRI_2_ – FM_2_	0.52	0.1	< 0.001
** Cross-lagged paths**			
MPQ-PRI_0_ → FM_1_	0.55	0.2	0.02
MPQ-PRI_1_ → FM_2_	0.36	0.1	0.001
FM_0_ → MPQ-PRI_1_	0.12	0.2	0.37
FM_1_ → MPQ-PRI_2_	0.12	0.1	0.18

*N* = 104

*β* standardized estimate, *FM* Fatigue Measure, *MPQ-PRI* McGill Pain Questionnaire-Pain Rating Index, *SE* standard error. Subscripts: 0 = pre-treatment, 1 = mid-treatment, 2 = post-treatment

Random intercept cross-lagged panel model (RICLPM) was used to estimate path coefficient. Variance and covariance of random-intercepts for the MPQ-PRI were set to zero because random-intercepts of MPQ-PRI were not significant; there was no effect of between-person (trait-like) for the MPQ-PRI. Model fit indices: comparative fit index (CFI) = 1.00, Tucker-Levis index (TLI) = 1.00, root mean square error of approximation (RMSEA) < 0.001 (90% confidence interval: 0.00–0.15), standardized root-mean-square residual (SRMR) = 0.03

There were significant cross-sectional associations between pain severity and fatigue symptoms at time of admission and post-treatment at a within-person level (β = 0.59, *p* < 0.001, and β  = 0.52, *p* < 0.001). The autoregressive paths for pain severity through the course of treatment were significant at pre- to mid-treatment and mid to post-treatment (β  = 0.60, *p* < 0.001 and β  = 0.65, *p* < 0.001, respectively), indicating that within-person deviations of pain severity from expected scores (i.e., the mean) predicted deviations from expected scores at the next time point. On the other hand, the autoregressive paths for fatigue symptoms through the course of treatment at the within-person level were not significant. Within-person cross-lagged paths from pain severity to fatigue symptoms were significant at pre- and mid-treatment (β  = 0.55, *p* = 0.02) and mid- and post-treatment (β  = 0.36, *p* = 0.001); pain severity predicted fatigue symptoms. There were no significant cross-lagged effects from fatigue symptoms to pain severity; fatigue symptoms at pre- and mid-treatment did not predict pain severity at the next time point (mid- and post-treatment). The overall model fit of the RI-CLPM was good, CFI = 1.00, TLI = 1.00, SRMR = 0.03, and RMSEA =  < 0.001 (90% CI, 0.00–0.15).

## Discussion

The results of the study are consistent with previous research showing that scores on a measure of pain intensity are significantly correlated with scores on a measure of fatigue [1]. The findings of the present study extend previous research in showing that, through the course of a standardized risk-targeted behavioral activation intervention, early treatment changes in pain predicted later changes in fatigue; but early treatment changes in fatigue did not predict later changes in pain intensity. The findings do not support a bi-directional relation between pain and fatigue.

## Temporal relation between pain and fatigue

The results of systematic reviews suggest that fatigue is a common symptom reported by individuals with persistent pain conditions [1, 26]. Several prospective studies have reported that changes in pain precede changes in fatigue [8, 27, 28]. For example, Feuerstein et al. [8] reported that changes in pain intensity were prospectively associated with changes in the severity of fatigue in patients with low back pain. Similar findings were reported by Nicassio et al. [27] in a sample of patients with fibromyalgia. The findings of Feuerstein et al. [8] and Nicassio et al. [27] suggest that the functional linkage between pain and fatigue is proximal in time; in both studies, variations in pain significantly predicted next-day pain. Christie et al. [29] examined the time-lagged relation between pain and fatigue in patients with rheumatoid arthritis over a one-week period. Their findings suggested that pain prospectively predicted later fatigue only in a subset of patients. For the majority of patients, the relation between pain and fatigue was synchronous.

Several different mechanisms have been proposed to account for a temporal relation between pain and fatigue. Resource depletion accounts have been proposed where it has been suggested that the physical or psychological energy required to manage or cope with persistent pain ultimately depletes energy resources contributing to the experience of fatigue [30]. Olson and colleagues have conceptualized fatigue as a component of the stress response [31]. Van Damme et al. [10] suggest that pain-related fatigue is the subjective experience of disengagement from goal pursuits that occurs when the costs of pursuing activity goals exceed the benefits. The disruptive effects of pain on sleep have also been discussed as contributing to pain-related fatigue [32].

Research has also pointed to a number of physiological processes that might give rise to fatigue associated with pain. It has been suggested that systemic inflammation might underlie the association between pain and fatigue [33, 34]. For example, the inflammatory phase accompanying musculoskeletal injuries of the spinal column induces proinflammatory cytokines implicated in the multifidus muscle remodeling. These changes can lead to muscle fatigue as replacement of muscle fibers by adipose tissues weakens the muscle and decreases muscle endurance [35]. Other potential mediators of the relation between pain and fatigue include reductions in heat shock protein (HSP), brain-derived neurotrophic factor (BDNF) [36] and lower dopamine levels [37].

## Temporal relation between fatigue and pain

Fewer studies have provided support a prospective relation between fatigue and pain. Studies examining the trajectory of symptoms reported by primary care patients have revealed that symptoms of fatigue prospectively predict the later emergence of pain symptoms [11, 12]. In a community health survey of high school students, Siivola et al. [11] reported that participants who reported symptoms of fatigue were more likely to have developed a musculoskeletal pain condition when followed up 7 years later. Halder et al. [12] reported that high scores on a measure of fatigue predicted onset of abdominal pain in a community sample of adults when re-assessed 1-year later. Aili et al. [32] reported that symptoms of fatigue pre-dated the onset of pain in a large sample of individuals who later (5 years) developed chronic widespread pain.

Our failure to replicate previous findings showing a temporal relation between fatigue and pain might be due of several factors. First, the time frame of our study might not have been adequate to capture the time-dependent nature of the relation between fatigue (as a causal factor) and pain. Studies that have yielded findings supportive of a temporal relation between fatigue and pain have had follow-up periods ranging from 1 to 7 years. The timeframe of the present study extended only over a 10-week period. As well, studies reporting evidence of a temporal relation between fatigue and pain sought to determine the role of fatigue symptoms as a prognostic indicator of the onset of a pain condition at a later point in time. The present study addressed whether early treatment-related reductions in fatigue predicted later reductions in pain. It is possible that the mechanisms that underlie the prognostic value of fatigue for the onset of a future pain condition differ from those that are associated with treatment response.

It is important to consider that the analytic procedures used in all studies supporting a temporal relation between fatigue and pain have used group-based procedures and have not controlled for within-person associations. In all studies, the analytic approach involved the use of logistic regressions using categorized groups of fatigue with a binary outcome (presence of pain or not) to calculate odds ratios. As noted earlier, group-based procedures are associated with a higher rate of Type I error. In support of this explanation, when the data from the present study were analyzed using traditional CLPM, which does not control for within-person variance, results supported a bi-directional relation between pain and fatigue. However, when analyzed using RI-CLPM procedure, controlling for within-person variance, the paths from fatigue to pain were no longer significant.

## Clinical perspectives

Pain is an important determinant of magnitude of disability associated with musculoskeletal conditions [38]. Emerging research suggests that fatigue is a symptom that frequently co-occurs with musculoskeletal pain, and further adds to the burden of disability [39, 40]. Indeed, research shows that treatment-related reductions in fatigue prospective predict resumption of occupational activities in individuals with musculoskeletal conditions [24]. Surprisingly, there is a paucity of research that has addressed how best to manage debilitating symptoms of fatigue to promote rehabilitation progress in individuals with musculoskeletal conditions.

In the present study, participation in a behavioral activation intervention was associated with a 20% reduction in fatigue, corresponding to a moderate effect size (*d* = 0.66). The effect size was higher when analyses were conducted only on the subsample of participants who initially presented with severe (> 6/10) symptoms of fatigue (*d* = 0.71). In previous research, reductions in fatigue of 20% have been shown to be sufficient to translate into meaningful clinical outcomes such as return to work [24].

On the basis of the findings of the present study, it is possible to speculate that early-treatment reductions in pain might contribute to reductions in fatigue in the rehabilitation of musculoskeletal injury. In turn, reductions in fatigue might promote fuller engagement in treatment, and contribute to more positive rehabilitation outcomes [24]. It is not clear however, that all techniques, modalities or medications that reduce pain severity will also be associated with reductions in symptoms of fatigue in individuals with musculoskeletal conditions. It will be of interest to determine whether other treatments typically used in the management of chronic pain, such as medication, heat/ice, laser, electrical stimulation or relaxation also prospectively predict reductions in fatigue.

Emerging findings call for more attention to fatigue in the assessment and treatment of individuals with persistent pain conditions. Fatigue has been shown to contribute to disability in individuals with persistent pain conditions independent of the severity of pain symptoms [18]. Even though fatigue might be, at least in part, functionally related to pain severity, over time, fatigue might become self-sustaining. Prolonged activity withdrawal resulting from fatigue might contribute to an excessively sedentary lifestyle further complicating the clinical presentation by increasing the probability of developing medical and mental health co-morbidities [41, 42]. Interventions designed to target both pain and fatigue might be associated with better clinical outcomes than targeting either variable in isolation.

Some degree of caution must be exercised in the interpretation of the study findings. First, data records were drawn from the clinical files of individuals referred to an occupational rehabilitation service. Only a minority of individuals with debilitating pain conditions are referred for occupational rehabilitation services. In addition, all participants were receiving wage-replacement benefits. These sample characteristics necessarily have implications for the generalizability of findings. The modest sample size also limited the nature of analytic procedures that could be applied to the data. As well, it was not possible to examine the possible mediating or moderating role of other variables that could potentially influence the relation between pain and fatigue (i.e., type of medication, sleep, co-morbid health or mental health conditions). It is important to note that many participants in the present study were receiving other concurrent treatments such as medication or physical therapy. As such it is not possible to unambiguously attribute changes in pain and fatigue to the behavioural activation intervention.

## Conclusions

In spite of these limitations, the results of the present study suggest that there is a temporal relation between symptoms of pain and fatigue in individuals with musculoskeletal conditions. To our knowledge, this is the first study to show that, during a rehabilitation intervention, early changes in pain severity predict later changes in fatigue. Early treatment reductions in pain severity might contribute to reductions in fatigue, and in turn, foster fuller participation in rehabilitation interventions. More research is needed to better understand the pathways underlying the temporal relation between pain and fatigue. More research attention also needs to be directed toward the development of more effective means of reducing fatigue in individuals with chronic musculoskeletal pain.

## Data Availability

The datasets analysed for the current study are not publicly available since they are undergoing further analysis; but they are available from the corresponding author upon reasonable request.
